# Overview of Mobile Communications in Colombia and Introduction to 5G

**DOI:** 10.3390/s23031126

**Published:** 2023-01-18

**Authors:** Alexis Barrios-Ulloa, Dora Cama-Pinto, Francisco Manuel Arrabal-Campos, Juan Antonio Martínez-Lao, José Monsalvo-Amaris, Audomaro Hernández-López, Alejandro Cama-Pinto

**Affiliations:** 1 Department of Electronics Engineering, Faculty of Engineering, Universidad de Sucre, Sincelejo 700001, Colombia; 2 Department of Computer Architecture and Technology, University of Granada, 18071 Granada, Spain; 3 Faculty of Industrial Engineering, Universidad Nacional Mayor de San Marcos, Lima 15081, Peru; 4 Department Engineering, University of Almeria, 04120 Almería, Spain; 5 CIAIMBITAL Research Center, CeiA3, University of Almería, Carretera Sacramento, s/n La Cañada, 04120 Almeria, Spain; 6 CIMEDES Research Center, CeiA3, University of Almería, Carretera Sacramento, s/n La Cañada, 04120 Almeria, Spain; 7 Department of Computer Science and Electronics, Faculty of Engineering, Universidad de la Costa, Barranquilla 080002, Colombia; 8 Ericsson Latam North, Ericsson, Mexico City 02230, Mexico

**Keywords:** 4G, 5G, Colombia, mobile communication networks, mobile operators, spectrum

## Abstract

The deployment of 5G around the world continues to progress at a rapid pace, especially in North America and Asia. Its advantages and efficiency as a data transmission network have been widely demonstrated in different fields such as agriculture, education, health, and surveillance. However, this process does not have the same dynamics in Latin America, specifically in Colombia. The country is currently implementing actions aimed at facilitating the deployment of this technology in the short term, including pilot tests for the use of the radio spectrum, spectrum auctions, the planning of future auctions, and the review of spectrum caps. The results of this review allow us to conclude that despite the forecasts and the intentions of the Colombian government and mobile communication service operators, 5G in standalone mode will not be commercially available in Colombia before the end of 2023. The main failures in its deployment are related to the lack of available spectrum to support the ultrahigh-reliability and low-latency, enhanced mobile broadband, and massive machine-type communications scenarios, as well as the delay in the auction processes for its assignment.

## 1. Introduction

Ericsson’s Mobility Report 2021 records 8.2 billion mobile subscriptions worldwide, including all major wireless technologies (CDMA, GSM, HSPA, LTE, and 5G) [1]. The same report shows that second-generation (2G) and third-generation (3G) connections are shrinking every year, and estimates that the use of fourth-generation (4G) technologies will peak in 2021, a year with the rapid growth of fifth-generation (5G) networks. In Latin America and the Caribbean, the trend is towards a decrease in 2G and 3G networks, while 4G and 5G networks are on the rise, like the situation in the rest of the world. Therefore, 5G Americas forecasts indicate that from 2022 onwards, mobile connections via long-term evolution (LTE) will be decreasing, contrary to what will happen with 5G [2]. In the case of Colombia, the country has the third-largest population in Latin America [3] and one of the leading economies in the region [4], which is reflected in several indicators, including mobile telephony penetration, which reaches a rate of 70 per 100 inhabitants. [3]. For these reasons, it is important to know the current scenario of the mobile telephony market in Colombia since it plays an important role in the economic and social development of the country.

This research details the cellular frequency bands operating in Colombia associated with their economic auction values and the companies that own them. It also identifies key technical issues and highlights the state’s regulatory policy about mobile telephony. According to the academic literature reviewed, this is the first time that research of this type has been conducted, contributing not only to accelerating the implementation of the 5G telephony network that has not yet been offered commercially in Colombia but also by contextualizing national and international actors in this field of telecommunications from both industry and academia as a basis for research in the area of mobile technology and the socioeconomic impact of the country.

### 1.1. Current Networks

**Figure 1:**
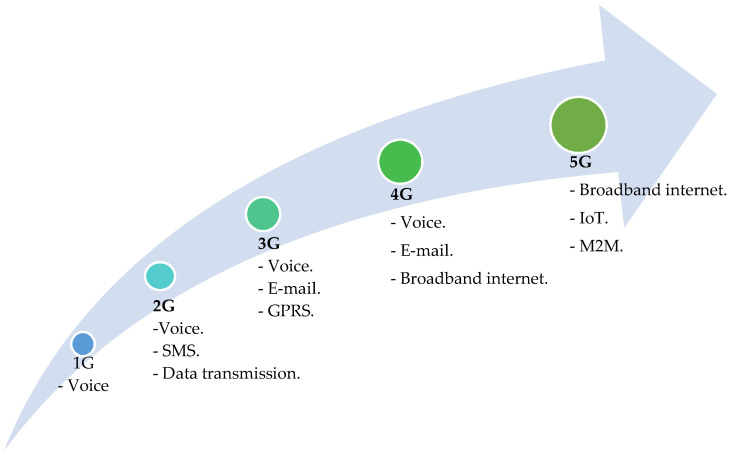
Evolution of mobile communications networks [5].

Mobile wireless communications have undergone a rapid evolution over four decades, starting in the 1980s with the first generation (1G), based on analog systems, to the current fifth-generation (5G), which will occupy a dominant position on the global horizon over the current decade, while the next will be 6G, which is expected to be available from 2030 [4]. Colombia is no stranger to this transformation of the telecommunications industry and currently has 2G, 3G, and 4G networks deployed in its territory. In the case of 5G, the state has launched a set of actions to enable its implementation in the short term. Figure 1 shows the evolution of mobile communications with the main milestones of each generation, and a comparison of the advantages and disadvantages of each is presented in Table 1.

Data from the Colombian Ministry of Information and Communications Technologies (MinICT) indicate that in 2019, close to 9.5 million users, corresponding to 31% of the total, accessed mobile telephony services with 2G and 3G technologies [11]. The technology of 2G is extremely limited in mobile data transfer, focusing on voice and text messaging services, which are the most common ways to connect to the network in the most remote areas of Colombia, mostly characterized as rural scenarios. The Colombian Chamber of Information Technologies and Telecommunications (CCIT) estimated in 2019 that approximately 10 million users in Colombia only had access to the mobile network through 2G, being one of the few countries in the world where this type of network is in operation [12]. The lack of high-speed connectivity hurts information access and competitiveness indicators in the most remote and backward areas of the country.

Completing the total “switch-off” of 2G and 3G as soon as possible is necessary and inevitable in Colombia for its economy and population to continue to evolve in the digital society, where mobile networks play a fundamental role in the exchange of information, including that generated from millions of data obtained from monitoring and measurement systems, such as the Internet of Things (IoT). In addition to the superiority of 4G and 5G in terms of capacity and data transfer speed, the disconnection of 2G and 3G will free up significant portions of the radio spectrum that could be used in more efficient systems (4G and 5G). For these reasons, in June 2020 the MinICT launched the Plan for Transition to New Technologies, which presents strategies to modernize the ecosystem of mobile communications services in Colombia [11]. In addition, the ICT Plan 2018–2022 aims to migrate 10 million users to access the Internet through 4G before the end of 2022 [13].

Following government guidelines, mobile telephone operators providing services in Colombia began the migration process. In this regard, Tigo-UNE informs that by the end of October 2022 it will close its 2G network [14]. Claro plans to dismantle its 2G network by December 2022 at the latest, concentrating its investments in the 4G network and the future 5G network [15]. Other operators, such as Movistar and Avantel, do not provide information on the 2G closure process. However, these companies are expected to carry out the process in harmony with MinICT guidelines.

**Figure 2:**
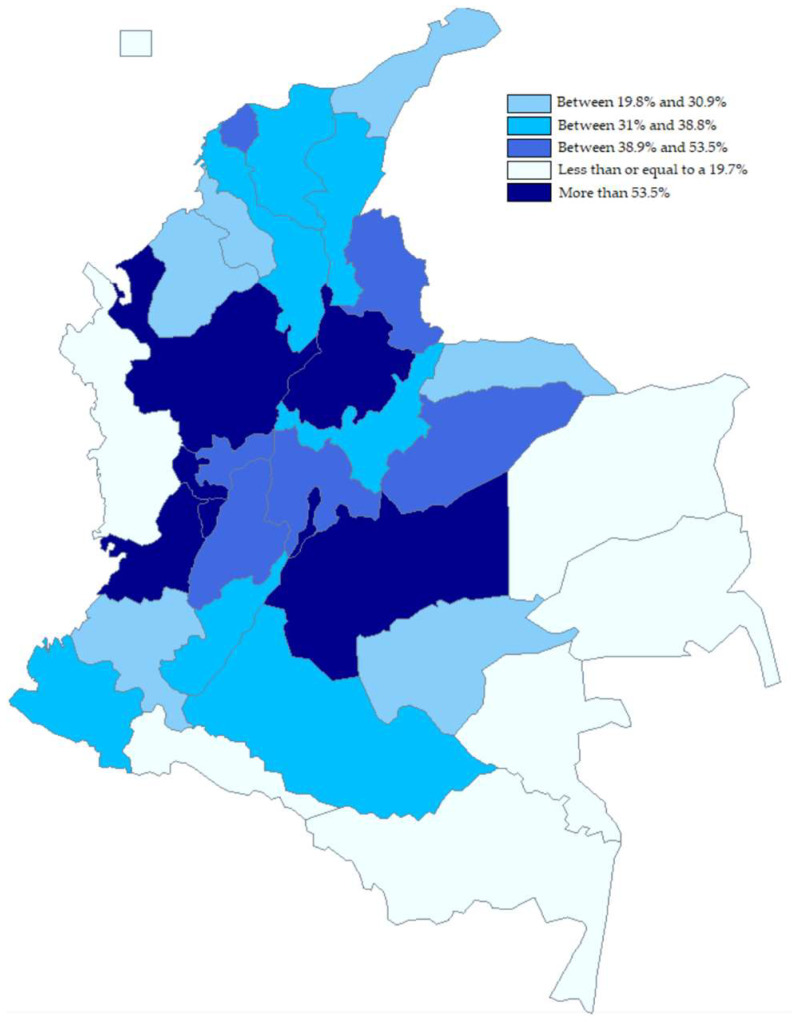
Proportion of households with Internet connection. Source: DANE [20].

Today, mobile communications go beyond simple person-to-person interconnection, allowing users to perform multiple tasks through cellular devices. However, the deployment of mobile networks does not exclusively affect human communication; they have also become an important tool for change in the development of different sectors of the economy. Connectivity and high-speed connection rates (especially through mobile devices) will become increasingly important in people’s daily activities and in the operation of businesses and institutions. In this context, IoT represents a business opportunity for cellular network operators, as IoT devices often need to connect over wide area networks to enable their application in different scenarios. Since the concept of IoT was introduced to consumers in 2009, predictions about its evolution have been coming true [16], and as a result of this development, IoT devices are connecting in smaller numbers via 2G or 3G networks, and since 2019, connections via technologies such as Narrow Band IoT (NB-IoT) or CAT-M are increasing [17]. Projections presented in [18] foresee some 27 billion connected IoT devices worldwide. Thus, populations, businesses, or institutions that are not connected or have limited coverage are more likely to be excluded from access to mobile networks, which reduces their quality of life [19]. In this context, Colombia is lagging in 4G coverage, and a large part of its territory (especially in rural areas) is covered by 2G and 3G networks. Although more than 80% of households in most of the departments that make up Colombia’s political–administrative structure have access to cellular telephony, in most of its territory, the percentage of households with Internet access is less than 53% (see Figure 2) [20].

Despite the fact that mobile telephony penetration in Colombia is high (131.6% approx.) [11], 2G and 3G coverage account for a large part of the coverage (31%). This last statistic indicates a significant disadvantage for the country when compared to Europe (21%), North America (9%), and China (2%). However, the situation improves when compared to North Asia (50%), Asia-Pacific (34%), and North Africa (58%). In Latin America, the percentages for Colombia are similar to those of the rest of the region (31%) [19]. It is undeniable that the increase in coverage and quality of mobile telephony services contributes to the social and economic growth of the states. For this reason, if Colombia is to have a digital society and economy framed by the industry 4.0 concept, it is important to move quickly to replace the current 2G and 3G networks in favor of 4G and 5G.

There is no consolidated information on the average speed rates of downstream and upstream channels of all operators providing service in Colombia, much less by region or department. Possibly because these providers do not offer broadband services, i.e., they do not guarantee a minimum speed and focus on offering per-download plans for some time (usually for a calendar month). However, statistics reported by MinICT in its fourth quarterly report for 2021 show a growing trend in mobile subscription Internet access. In addition, the average traffic in this type of access has also increased in the last two years, demonstrating the inclination of subscribers towards the use of mobile systems as an Internet access technology [21]. This growth is supported by several factors, such as increased speed, increased coverage, and lower tariffs.

### 1.2. 5G in Colombia

**Figure 3:**
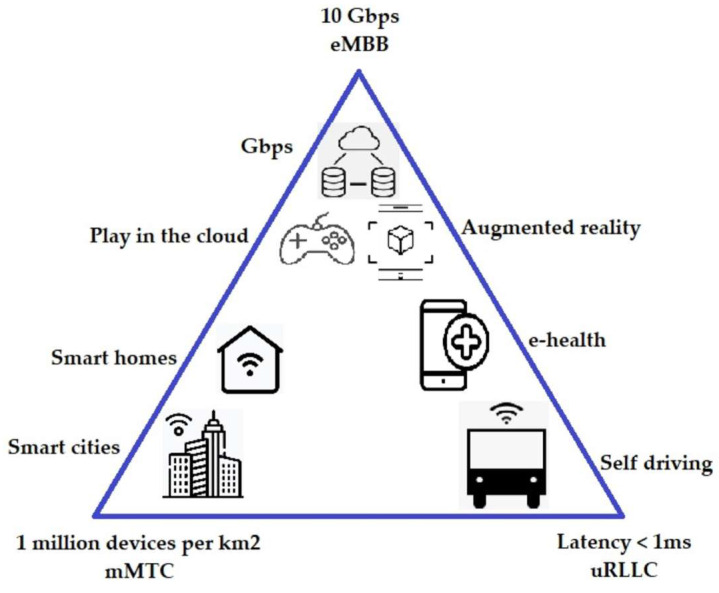
5G application scenarios.

Fourth-generation mobile systems offer enormous advantages over their predecessors, especially in terms of Internet connection speed. However, these networks are not capable of meeting the service needs of natural and industrial users. For example, they do not allow the connection of millions of devices that will be connected to the IoT. Some of the improvements that 5G has over 4G include 100 times faster data transmission, lower end-to-end latency, and higher capacity [22]. Another novelty of 5G is the variety of scenarios that are envisaged and that are related to different applications: enhanced mobile broadband (eMMB), massive machine-type communications (mMTC) and ultrareliable and low-latency communications (uRLLC). As for eMMB, it achieves mobile broadband thanks to high data rates, while uRLLC guarantees connection reliability thanks to low latency [23]. On the other hand, mMTC connects millions of devices (IoT elements) in a small geographic area [24]. Figure 3 presents the 5G application scenarios.

Of the three application scenarios, eMBB and uRRLC require more channel capacity than 4G. Since the radio spectrum below 1 GHz and from 1 to 6 GHz is saturated with wireless services at some intervals, a portion of the centimeter waves (above 6 GHz up to 30 GHz) and millimeter waves (between 30 and 300 GHz) will enable the bandwidth needed in 5G, as is the case with eMMB aimed at offering high-speed, low-latency services. To this end, the United States auctioned spectrum blocks at 24, 28, 37, 39, and 47 GHz [25], and Brazil and Sweden at 26 GHz [26,27]. The most prominent challenge to overcome in the efficient deployment of 5G using the centimeter and millimeter bands is the increase in radio signal attenuation as the frequency increases, requiring rigorous planning of the radio infrastructure.

The monthly global mobile data volume will increase fivefold by 2024 compared to 2018, reaching 136 exabytes [28]. This amount is partly high due to orthogonal frequency division multiplexing (OFDM), the dominant waveform in 4G and 5G wireless communication systems that increases the data rate [29]. The third-generation partnership project (3GPP) in the 5G new radio (NR) physical layer chose cyclic-prefix OFDM (CP-OFDM) as the waveform to support downlink (DL) and uplink (UL) transmission, while DL also uses DFT-s-OFDM (discrete Fourier transform spread based) in the frequency ranges of FR1 from 410 MHz to 7.125 GHz with bandwidth up to 100 MHz; and FR2 from 24.25 to 52.6 GHz with bandwidth up to 400 MHz [30,31,32]. DFT-s-OFDM closely resembles the single-carrier SC-FDMA waveforms used in LTE UL. DFT-s-OFDM, based on CP-OFDM, and operates DFT at the transmitter side to spread the signal among the allocated subcarriers, resulting in lower PAPR (peak to average power ratio) in coverage-limited user equipment (UE), because energy efficiency is a critical issue in transmissions for mobile users (MUs) battery [33,34]. For their part, the CP-OFDM coding technique adds the tail portion of each OFDM symbol to the header, generating a guard band with a duration equal to the expected delay [35]. In this way, it reduces inter-symbol interference (ISI) in channels where bit errors occur due to delays caused by different propagation paths [36].

The introduction of 5G is often facilitated by sharing part of the radio spectrum of 4G-LTE cellular networks with 5G NR. Thus, choosing CP-OFDM at the PHY layer makes it complement, coexist, and adapt to each other’s multiple similarities [37], even though LTE uses fixed 15 kHz subcarrier spacing for the network-wide data channel and 5G uses integral 15 kHz multiple subcarrier spacings given by 15 ∗ 2η (η integer) [38,39].

Fifth-generation technology increases channel capacity with more bandwidth per carrier (from 5 to 400 MHz) [40], being spectrally more efficient due to the higher orders of the modulation schemes [41,42]. Previously employed in 4G, the multiple-input multiple output (MIMO) technique simultaneously uses multiple antennas with multiple channels for signal transmission and reception, increasing channel bandwidth and maintaining antenna efficiency without requiring additional power [43,44]. Antenna gains in the MMIMO (massive MIMO) version used in 5G compensate for channel propagation losses by providing robustness against interference and low latency [45]. OFDM (orthogonal frequency division multiplexing) is another technique used in 5G to reduce the spacing between multiple subcarriers. However, the shorter the spacing, the longer the OFDM symbol duration, and the higher the communication latency [39].

**Figure 4:**
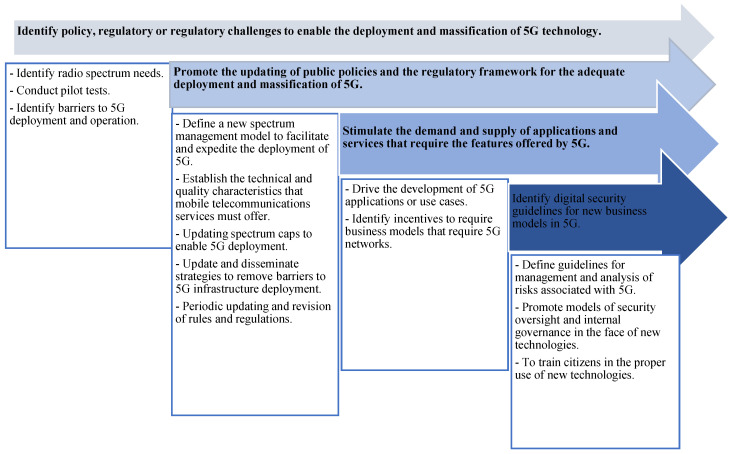
Specific objectives and lines of action of the 5G Plan. Source: [46].

Currently, no operator in Colombia offers 5G mobile communication services. However, the national government, through MinICT, has drawn up a roadmap for the transition from 4G to 5G. With this objective in mind, in 2019 they launched the 5G Plan, which defines the strategies that will allow for the implementation of these networks in the Colombian territory. Figure 4 shows the objectives of the 5G Plan, as well as the lines of action defined in each of them.

### 1.3. Objectives and Motivation

The main objective of this article is to present the current panorama of mobile telephony in Colombia, considering state policies and the strategies of the different market players. The specific objectives of this article are:

- To present the current state of mobile cellular systems in Colombia.
- To document the strategies to be implemented by the Colombian state and mobile operators for the massification of 4G networks and the introduction of 5G.
- To identify the situations that have prevented the implementation of 5G in Colombia.

This document will become a reference source for decision makers involved in the planning and implementation of mobile communication systems, not only in Colombia but also in other countries with similar situations in terms of telecommunications regulation and spectrum management. It will also be a roadmap for researchers who wish to carry out projects or intend to support communication or data transmission systems over cellular networks, allowing them to identify the weaknesses and strengths of the mobile telecommunications system in Colombia.

This document is structured as follows: Section 2 explains the methods of querying and filtering the information used as a reference in the article. Section 3 provides an analysis of the current state of 4G networks in Colombia, as well as the future of 5G and the factors that have prevented its implementation. Finally, Section 4 presents the conclusions.

## 2. Methodology

An exhaustive search in specialized databases did not find any articles that present in detail the current situation of the mobile wireless communications sector in Colombia, which makes this document a valuable tool for all those interested in using cellular networks as a communication or business strategy. Globally, the information found is more extensive and widely used as reference material, such as the Ericsson Mobility Report [47]. Some reports by the Global System for Mobile Communications Association (GSMA) also addressed the issue in a global and Latin American framework, but with a focus on 5G networks. For example, in [48] they set out the organization’s views on the use of radio spectrum, while in [49] they discuss the use of the 3.3 to 3.8 GHz frequency range for 5G in Latin America. As for the Colombian case, some review articles have presented analyses of the possibilities for the use of cellular networks in Colombia, but like the GSMA, they have focused on 5G [25,45,50].

The information used in this research comes from three sources. The first belongs to the documents on the websites of the state entities in charge of managing public telecommunications policy: MinICT, the National Spectrum Agency (ANE), and the Communications Regulatory Commission (CRC). In addition, queries were sent to these bodies via the e-mail addresses that have been set up for citizens to request different types of information. The second source of information corresponds to the information published by private organizations (operators, associations, and collectives) and is available through their websites of each of them. The rest of the information was extracted from scientific articles, conference papers, books, and book chapters resulting from the SCOPUS database search. The terms used in the search strings were: “2G”, “3G”, “4G”, “5G”, “agriculture”, “Colombia”, “education”, “frequency bands”, “health”, “MIMO”, “mobile operators”, “mobile telecommunications networks”, “OFDM”, “policy”, “security”, “spectrum”. This consultation was limited to its initial appearance in the titles, abstract, and keywords. To further refine the large number of documents available, additional filters were used in the selection of the information, which can be found in Table 2.

Finally, the information collected was analyzed and recorded. In total, 105 references were used as sources for this article. A total of 32 correspond to documents or web pages of Colombian state entities (e.g., MinICT, CRC and ANE), 6 come from associations or organizations formed by different countries (United Nations, multilateral banking, ITU), 19 correspond to reports or web pages of private entities (manufacturers, operators, and private associations), and 54 correspond to documents extracted from the SCOPUS search.

## 3. Analysis and Results

### 3.1. Current Networks

The telecommunications sector in Colombia is regulated by the provisions of Law 1341 of 2009. This regulation aims to promote access to information and communication technologies (ICT) to achieve universal service as well as increased connectivity. It also ensures, inter alia, the efficient deployment and use of communications infrastructure and the efficient management of the radio spectrum [51]. To update the regulations, Law 1978 of 2019 was formulated, with provisions that modernize the ICT sector [52]. These laws frame the procedure for assigning the radio spectrum to telecommunications operators. Furthermore, spectrum management in Colombia is in line with the International Telecommunication Union (ITU) in terms of the economic aspects derived from the exploitation of this activity, as set out in recommendation ITU-R SM.2012-6, updated in 2018 [53]. In this regard, the regulation allows the use of the radio spectrum for an initial defined period of 20 years, which can be renewed for up to 20 additional years to allow operators to adequately plan their short-, medium-, and long-term investments in infrastructure for mobile and data services. In other words, with the confidence granted by the concession period, the prolonged operation is promoted based on significant investments with a higher chance of return.

The radio spectrum is a limited and valuable natural resource in any region of the world, and countries develop policies that allow them to use it rationally and efficiently, maximizing the benefits obtained from its exploitation. The portion allocated to mobile communications systems is possibly one of the most coveted within the set of bands that make up the radio spectrum, as demonstrated by the values achieved in the different auctions (see Table 3).

Table 3 shows the high costs bid by different operators in the 2019 4G auction for access to the 700 MHz band. Although the subgigahertz bands have a lower bandwidth capacity, they are very attractive because of the ease of radio wave propagation, which allows for lower transmission infrastructure costs by providing coverage over a geographical area with fewer base stations (BS) compared to the higher bands. This is also evident in Table 3 when comparing the values offered by the 2.5 and 2.6 GHz bands. However, if one compares the prices and amounts of the spectrum allocated to these bands in 2019 with what happened in 2013, there is a greater demand for the spectrum given the growth of the market and the current needs of users. As a result, the various stakeholders agreed to make higher outlays to obtain use permits. In addition, higher frequency bands (centimeter and millimeter waves) allow more of the spectrum to be made available, which facilitates the deployment of specific applications for the eMBB scenario.

As for spectrum licenses in the 850 MHz bands, these were not granted through auctions, as the regulations in force before 2009 allowed for their award through competitive bidding processes [54]. Currently, this portion of the spectrum is 100% allocated until March 2024, with a total of 50 MHz of bandwidth distributed as follows: 25 MHz to Colombia Telecomunicaciones and 25 MHz to Comunicación Celular S. A. (Comcel) [55], [56]. In addition, it is important to note that 22 MHz are available between 894 MHz and 905 MHz and between 939 MHz and 950 MHz [57].

Until June 2022, permits for the use of radio spectrum within the bands allocated to mobile services in Colombia are concentrated in five companies. However, current regulations allow for the provision of mobile telecommunications services without the need for licenses to use the bands allocated for this purpose, through the mobile virtual network operator (MVNO) modality. There are two categories of MVNOs in Colombia: resellers and full MVNOs. In the case of resellers, they are exclusively engaged in the sale of voice services on a prepaid basis, purchased from any of the mobile operators with infrastructure, and offering wholesale prices. Full MVNOs have a more advanced billing service and their switching infrastructure, and only lease the radio transmission service, which allows them to offer additional services [58]. Regardless of the modality, MNOVs have been driven by the need to increase competition for the benefit of users. Table 4 shows the operators offering the service in the two categories.

**Figure 5:**
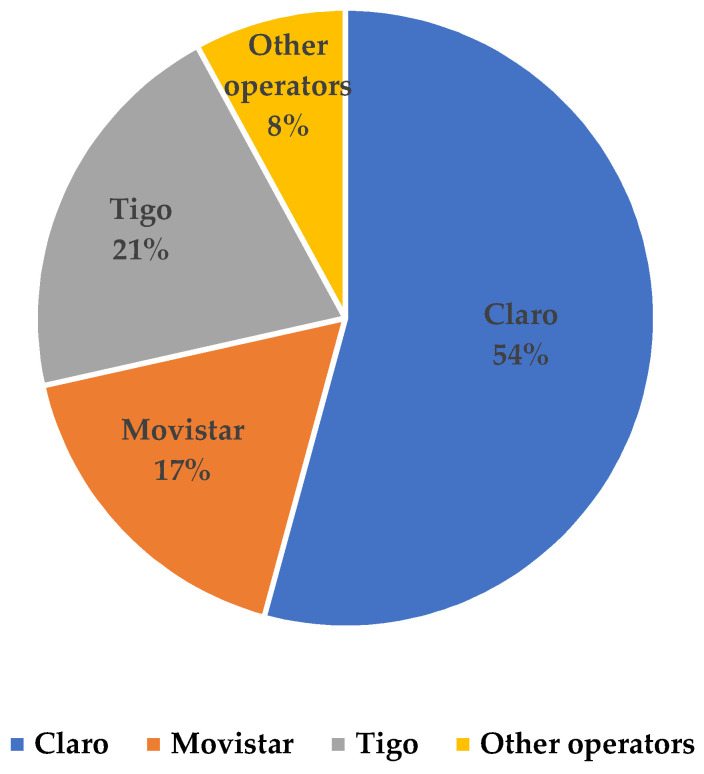
Percentage of mobile Internet accesses by subscribers in 2022. Source: [60].

**Figure 6:**
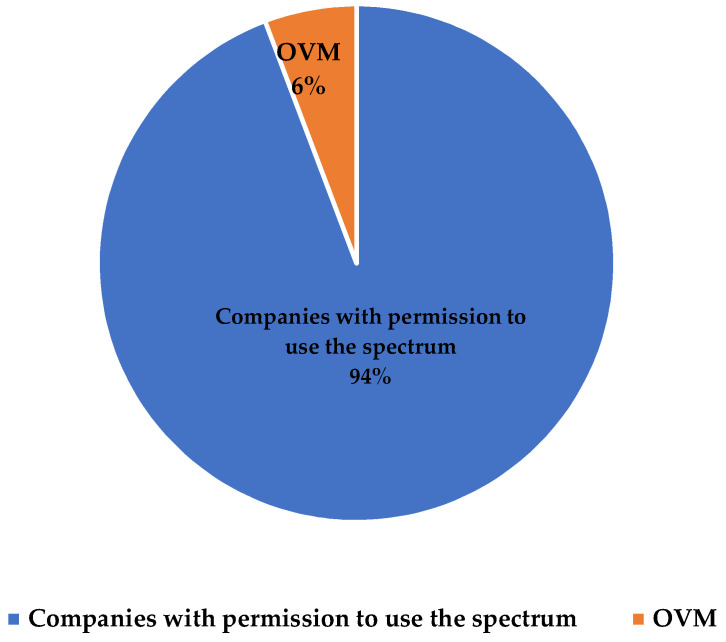
Percentage of traffic carried by mobile operators in 2022. Source: [60].

Although MVNOs have been offering the service in Colombia for several years and usually at relatively low tariffs, they have not been able to gain a significant market share. This may be due to several factors, including (1) limited capacity to offer broader services (lower speed or download rates), (2) little or no integration with other telecommunications services (TV and fixed internet), and (3) limited or no capacity in infrastructure operation and planning. The possibility of being able to use spectrum in large proportions is an advantage when offering mobile telecommunications services. Figure 5 shows that in 2022 the market share of the operators with the largest amount of allocated spectrum (Claro, Tigo, and Movistar) is significantly higher compared to the rest of the operators that have fewer spectrum resources or offer services through MVNOs. Furthermore, in the same period, of the total mobile internet traffic in Colombia (448,046 GB in total), only 6% was carried via MVNOs, while the remaining 94% was carried by licensed spectrum companies (see Figure 6). These data show that incumbent operators have a very large market share, which should motivate government agencies to develop policies that allow MVNOs to become more competitive for the benefit of users.

**Figure 7:**
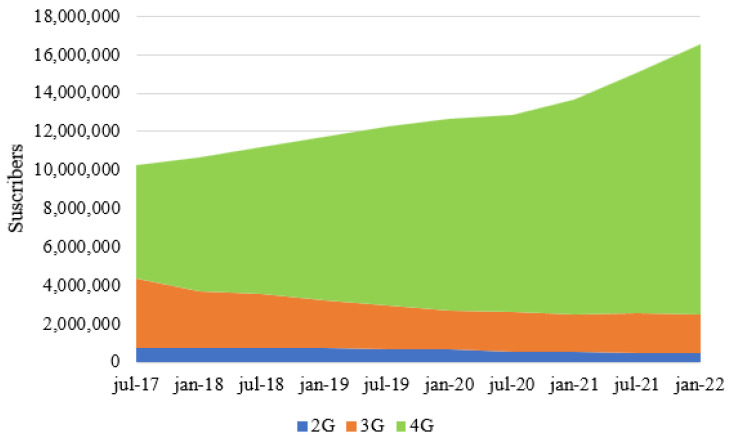
Mobile subscribers in Colombia by technology in 2022. Source: [60].

In terms of mobile Internet access, there has been a notable increase in demand for 4G technology. As shown in Figure 7, there is a decreasing trend in the number of users who used 2G and 3G as their access methods. In addition, a spike in 4G access was observed from July 2020, a period that coincided with the social isolation measures resulting from the SARS-CoV-2 virus (COVID-19), which forced millions of people to carry out different activities through remote or virtual access.

**Figure 8:**
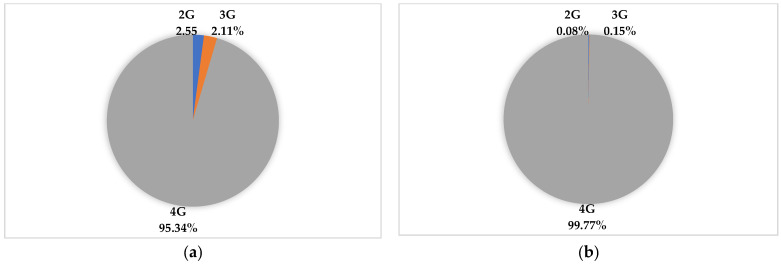
Technologies by BSs: (**a**) municipalities with fewer than 100,000 inhabitants and (**b**) municipalities with more than 100,000 inhabitants. Source: [61].

Colombia has made great strides in the modernization of mobile telecommunications networks, especially in the deployment of 4G in large and small cities. Figure 8a,b present information on the technologies installed in the BSs deployed in municipalities with populations over and under 100 thousand inhabitants.

The MinTIC's projections for the modernization and expansion of cellular networks in Colombia indicate that this process should be carried out in the short term, prioritizing the definitive transition to 4G and the expansion of coverage in the national territory, especially in rural areas, based on the obligations acquired by the different operators in the spectrum auctions, especially for those that obtained permits for use and commercialization in the 700 MHz band. In 2020, 4G coverage in municipalities with less than 100,000 inhabitants is mainly concentrated in the Andean region, which is the richest and most industrialized area of the country. Although 4G coverage in Colombia will grow significantly, there will still be large areas of the territory without this service, especially in the south of the country, characterized by populations that have historically lagged in terms of information and communications technologies (ICTs) [62]. In this sense, the Colombian state will have to develop new strategies to reduce the digital divide that affects the most developed areas of the country.

### 3.2. Deployment of 5G in Colombia

**Figure 9:**
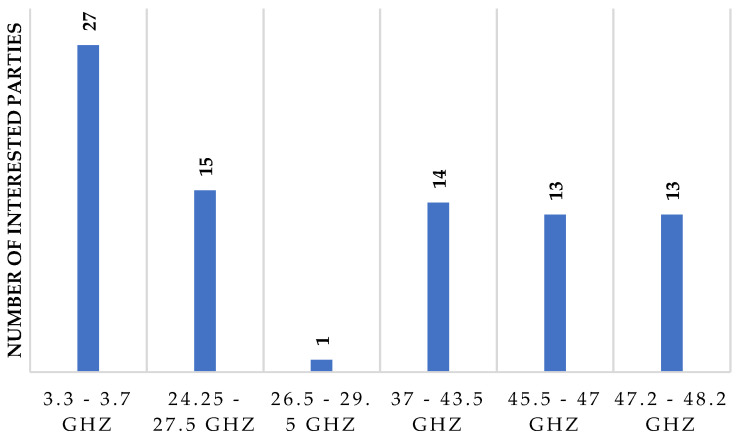
Interest in using radio spectrum for 5G pilot tests per frequency band. Source: [64].

As explained in Section 1.2, the MinICT has planned the implementation of 5G in Colombia through the official document called “Plan 5G”, which is the roadmap for the sector. Subsequently, there have been different actions aimed at the adoption of 5G in Colombia, and in that sense, in 2019, the MinICT invited those interested in conducting 5G pilot tests to express their interest in participating [63]. Finally, in 2020, the final list was published, consisting of 52 entities and 24 individuals (76 in total), interested in testing mainly in the 3.3 GHz to 3.7 GHz band (See Figure 9).

**Figure 10:**
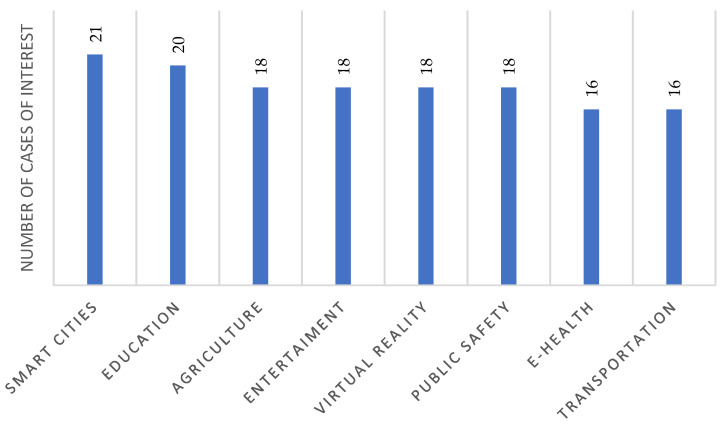
Use cases presented in the expression of interest for 5G pilots. Source: [64].

In terms of reported use cases for testing, there was interest in deploying 5G in different scenarios: smart cities, education, agriculture, entertainment, virtual reality (VR), public safety, health, and transport (see Figure 10). This demonstrates the projection of those interested in integrating 5G in different economic or social sectors in Colombia. A large number of permit applications and areas of application interest presented in Figure 9 and Figure 10 demonstrate that 5G is an attractive technology for rapid deployment in Colombia. In addition, the fact that many entities have expressed interest in frequencies above 24 GHz indicates that operators and other players in the mobile communications sector are aware of the potential of these bands due to a large amount of spectrum available, which could help the first 5G deployments in the country to match the eMBB scenario.

Subsequently, in April 2020, the MinICT issued Resolution 000638 [65] in line with the ITU recommendation in its document entitled “Laying the Foundations for 5G: Opportunities and Challenges 2018” [66], starting the process of granting temporary permits for the use of the radio spectrum for the exclusive purpose of conducting pilot tests in the range of 3.7 GHz to 23.6 GHz, 71 GHz to 76 GHz, and 81 GHz to 86 GHz. With these tests, different Colombian government agencies (MinICT, ANE, and CRC) and different operators aim to acquire a broader knowledge on aspects related to the efficient use of the frequencies considered in the study, as well as others related to the deployment of networks and the provision of 5G services. It is important to clarify that these spectrum use permits cannot be used for commercial activities and are valid for six months, extendable once for up to six additional months in accordance with the provisions of Resolution 467 of 2020 of the MinICT [67]. Finally, in July 2020, it granted permission for the use of radio spectrum for 5G trials to five telecommunications network and service providers (TNSPs) (see Table 5).

Table 5 shows that four of the winning TNSPs received use permits in the 3500 MHz to 3600 MHz range and one in the 3300 MHz to 3400 MHz range, which are part of the set of frequencies favored for the introduction of 5G deployments in Colombia and the rest of the world. One of the most important advantages of these bands is the greater amount of bandwidth available. However, as explained above, being higher than other frequencies used in mobile telecommunications services (e.g., 700 MHz, 800 MHz, 1800 MHz, 1900 MHz, 2100, and 2500 MHz) increases the problems of radio wave propagation and penetration into buildings, which makes it necessary to increase the power at the BSs or to deploy more of them to ensure coverage in each area. It is important to clarify that the provider Xiro Investment Group was also authorized to conduct tests in the 587 MHz to 592 MHz bands, which was not originally included in Resolution 000638 but was requested based on the regulations of Resolution 467 [35].

**Figure 11:**
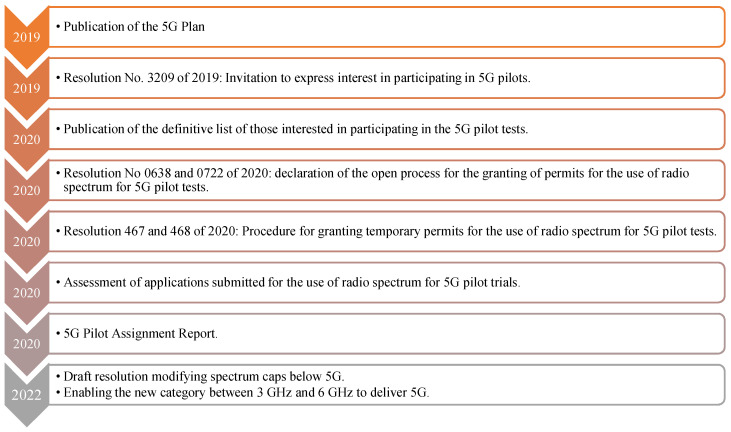
Actions implemented by the MinICT for the adoption of 5G. Source: [68,69].

The results of the executed tests have not been published and are still being conducted, not least because of the COVID-19 pandemic that started in 2020, with social, commercial, and legal restrictions that prevented their normal development. Currently, the MinICT, in collaboration with the ANE, is working on the future issuance of a Decree (an administrative act enacted by the executive branch) that will enable the development of 5G mobile services. The rule aims to increase spectrum caps in bands below 3 GHz, as well as to enable a new category between 3 GHz and 6 GHz for future 5G services. This decree will benefit 5G applications and scenarios requiring higher speeds [68]. As for spectrum auctions for 5G, in July 2022 there were no known dates, caps, bands, or economic aspirations on the part of state entities. However, in June 2021, the Vice Minister of Connectivity stated that they are working together with the ANE and the CRC on structuring the future auction to grant licenses to commercially offer 5G. Figure 11 shows the different processes implemented in the framework of the action plan.

### 3.3. Failures in 5G Deployment in Colombia

The advantages of 5G over previous technologies are considerable: faster download speeds, lower latency, and the ability to connect more devices [70]. Despite this, its implementation has not been easy at the global level, and even less so in Colombia. Ericsson, in its Global Mobility Report 2021 [47], indicated that the regions of the world with the highest number of 5G connections were North America and Northeast Asia, reaching only 20% and 24%, respectively, of total mobile connections. As for Europe, there is no significant growth, which contrasts with the economic power of this region, which is home to some of the world’s most important economies (e.g., England, Germany, France, and Italy). In this case, Western Europe accounted for 6% of 5G subscriptions, but projections indicate that by 2027 it could be the region with the second-highest number of 5G connections.

**Figure 12:**
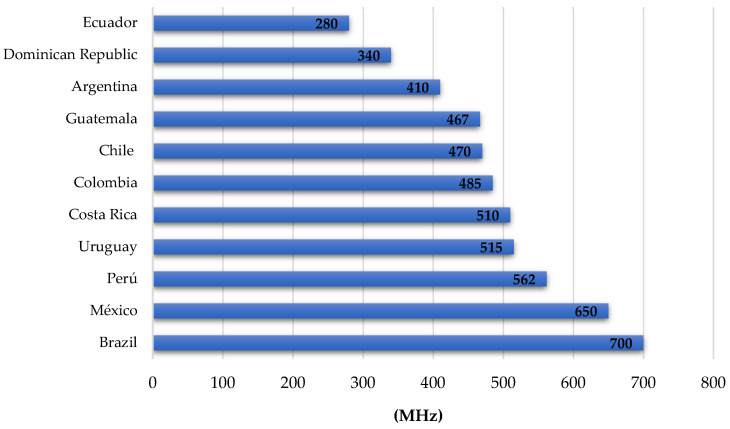
IMT spectrum in Latin America. Source: [49].

**Figure 13:**
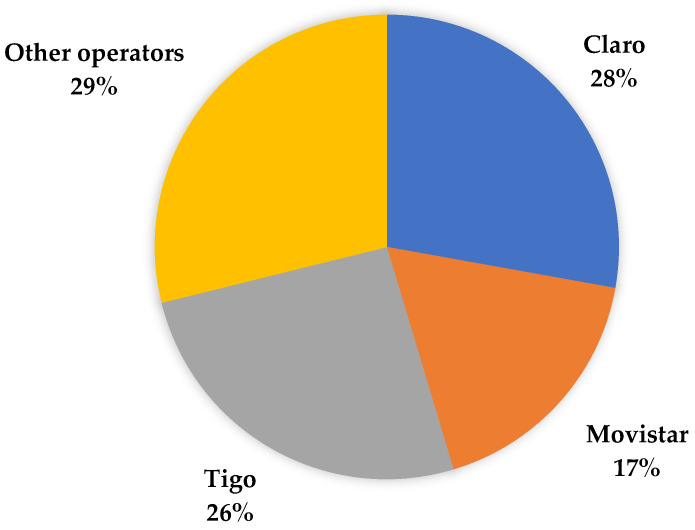
Percentage of IMT spectrum occupation by operators in Colombia. Source: [54].

Although standards such as Release 15 and 16 have already been approved [71], there is no unified view on what 5G can actually be defined as, and this is partly due to the particular interests of each of the actors involved in the mobile communications environment: regulators, consumers, technology manufacturers, service enablers, and connectivity providers, among others [72]. Some of the main issues associated with the deployment of 5G around the world relate to (1) people’s concerns about health impacts due to radio frequency emissions and the potential for 5G use in the future [73], (2) high infrastructure investment costs and forced changes of users’ terminal equipment [74] and (3) the number and type of radio spectrum bands needed for 5G [75]. In the case of Colombia, various situations have not allowed the concrete development of 5G in the country. One of the main drawbacks is the allocation of spectrum for commercial 5G developments, which causes delays, especially for operators, who cannot plan their network and acquire technology until this aspect is defined. In the Latin American context, Colombia ranks sixth, with an occupancy of 485 MHz for IMT (International Mobile Telecommunications) services, while the average in the region is 490 MHz [49,61], (See Figure 12). This situation undermines Colombia’s competitiveness, as investments in mobile telecommunications are large, and few companies can afford them. Proof of this is that three operators (Claro, Tigo, and Movistar) account for 71% of occupancy, while the remaining 29% is shared between three companies (Direct TV, Avantel, and WOM). In addition, the current spectrum caps are 90 MHz in the high bands and 45 MHz in the low bands [54]. Therefore, the limited availability of the resource and its concentration in a small number of providers limits the intentions of other companies to enter the Colombian market and make the necessary investments to increase coverage and offer modern services (see Figure 13).

In addition to the insufficient amount of IMT spectrum, assignment processes in Colombia are lengthy and slow to be structured due to their complexity and the substantial resources involved [51]. For this reason, in-depth and specialized (but lengthy) studies are carried out to ensure economic management that maximizes profit. In addition, there are the interests of incumbent operators seeking to make better profits and maintain their dominant position. In this regard, only four auctions have been held since the regulation was published (2010, 2011, 2013, and 2019). While the first three events took place over four years, it took six years to carry out the auction of 4G services, which is an important part of the future 5G rollout, as forecasts show that its initial deployment phase will be based on the nonstandardized architecture (NSA) used by the 4G-LTE network, allowing 5G to be implemented in less time using existing infrastructure [76,77]. Three years have passed since the 2019 auction and there is still no planned or estimated date for the 5G spectrum auction, adding to Colombia’s delay in its implementation.

As explained above, the NSA will facilitate the deployment of 5G, but without allowing access to the full benefits of standalone (SA). Therefore, it is expected that by the end of 2022 at least 80% of mobile Internet access will be via 4G [61]. Despite initiatives and expectations, 4G massification in Colombia has not been uniform, as not all municipalities have access to all operators’ networks and only one operator offers service in close to 99% of localities [61]. To improve this situation, companies that won spectrum in the 700 MHz band in the 2019 auction made commitments related to increasing coverage. MinICT Resolution 3078 of 2019, which regulated the process, obliges providers to upgrade 100% of municipalities that had 2G or 3G in 2019 to 4G technology within four years. However, there is no explicit obligation to extend coverage to localities that do not have any kind of mobile network, nor to extend their networks to all of them [78], and by 2025, the projected percentage of coverage will be 80% in rural areas [79], with a significant percentage remaining unserved.

### 3.4. Expectations for the Implementation of 5G in Colombia

The future implementation of 5G in Colombia allows the different participants in this hypothetical scenario to consider different uses. Although there is no commercial 5G infrastructure, there are reports of some applications that have been tested by PRSTs with permission to use the spectrum for 5G trials together with public and private organizations. For example, Claro defined three use cases in its pilot tests: mobile terminals via eMBB, fixed terminals for fixed wireless access (FWA), and a private 5G network located in a shopping center in the city of Bogotá [80]. Among the areas benefiting from these experiments is healthcare, which will benefit from 5G through the transmission to the cloud of the images resulting from computed tomography (CT) scans, corresponding to the uRRLC scenario. Additionally, in the educational field and in alliance with the Mayor’s Office of Medellín, a pilot project was carried out that allows students in educational institutions to develop classes with the support of cameras and high-definition video [81], which is in line with the characteristics of eMBB. Movistar also focused its 5G-related resources on telemedicine, facilitating the transmission of real-time video images, and allowing doctors to assess remotely located patients [82,83]. Tigo is one of the providers that has developed the most applications in the framework of the pilot tests, also in partnership with the Mayor’s Office of Medellín and Nokia, presenting seven 5G use cases: eMBB, FWA, navigation and assisted maintenance for drivers, collaborative robots, 360° VR, video analysis, and smart lighting [84], the latter framed in mMTC. Finally, Xiro Investment, through the IDTOLÚ Research and Development Lab, is focused on testing LTE-based 5G in audiovisual broadcasting (TV and radio) using the FeMBMS (further evolved multimedia broadcast multicast service) transmission mode [85]. In addition to the trials conducted by the PRSTs, different researchers in Colombia have been working on thematic lines related to 5G. For example, in [62], they simulated 5G radio signal propagation at 28 GHz, 38 GHz, and 60 GHz, while [63] performed simulations in the 3.5 GHz band, both in the city of Bogotá.

Pilot tests in different use cases carried out by TNSPs in Colombia demonstrate that 5G can become a tool to increase and strengthen the competitiveness of various economic sectors while helping to reduce the digital divide. Thanks to 5G’s improvements in speed, latency, and density of connected devices, it is possible to integrate other technological solutions that help improve everyday production or human processes. One of these is IoT, in which through the use of 5G, multiple sensors and software platforms will benefit the development of smart environments [86].

There is a field of opportunities in Colombia to implement 5G/IoT in different areas, including agriculture, livestock, tourism, industry, environment, and health [25], in addition to others such as education and smart environments implemented during the testing process, which are of special interest for the economic and social development of this country. In this respect, telemedicine is one of the areas that could benefit most from 5G, and proof of this is the interest of TNSPs in this sector during the pilot phase in Colombia, a country with many locations too far away from the main populated areas and where permanent medical services are rarely available. Although the concept of e-health is not new, 5G could leap in quality and expand the coverage of medical services thanks to the advantages over previous generations of mobile networks. In the case of telemedicine, the uRLLC scenario offers the necessary features of high transmission speed and low latency that are necessary for the timely diagnosis and treatment of diseases [87]. Another candidate sector for multiple 5G/IoT applications is agriculture, an activity of great importance for present and future food sustainability. Moreover, its operation requires many resources (water, soil, fertilizers, nutrients), which need to be optimized to reduce costs and minimize its impact on the environment. Modern agriculture is framed within the concept of Industry 4.0, and integrates tools such as artificial intelligence (AI), big data (BD), unmanned vehicles (UAVs), and IoT, among others [50,88], and all of them use large amounts of data in their operation, hence the importance of 5G in the communication phase. Like e-health, agriculture benefits from remote monitoring of variables, in this case related to the state of plants, soil, or climate [89]. However, it can also be used in the transmission of data to UAVs or in the control of irrigation systems, or in the management of commercial crops. As with medicine, it is difficult to provide quality education at any level in the most remote areas of Colombia. This is due to different factors, but perhaps the most influential are (1) geographical isolation, which is reflected in the quality test results of pupils in rural areas; and (2) the low interest of teachers with higher levels of education to serve in hard-to-reach areas [90]. In this sense, 5G opens up great possibilities as a tool to bring courses taught in urban areas and the best institutions to the most remote rural areas, reinforcing teaching mechanisms with remote access through ICTs [91]. Students in urban areas, especially in universities, are also expected to create new digital skills based on tools, including AI, BD, IoT, 5G, and VR [92,93]. The fifth-generation network is also an alternative communication network that enables the transmission of data to police, military, and other law enforcement agencies. Although security indices have improved in Colombia, the perception of insecurity among citizens is currently high, especially in urban areas, with a rate of 42.6 percent in urban areas and 26.3 percent in rural areas [94]. Public or private surveillance activities make use of 5G in some countries, benefiting from the high speeds enabled by the eMBB scenario and the large number of devices (e.g., video cameras) that are possible in mMTC. The use of 5G/IoT generates development expectations in different social and economic sectors in Colombia. However, its implementation poses challenges for operators and regulators, given the country’s geographical and socioeconomic conditions. For example, in 2024, when the commitments made by operators in the 2019 auction are fulfilled, there will still be a significant percentage of the territory (approximately 20%) without 4G network coverage, which would be the basis for the first 5G networks, and as these areas are very remote from urban centers and have a population with low purchasing power, the deployment of modern cellular telephone networks is not attractive due to the difficulties in recovering the investment. Therefore, there is a need to develop strategies to facilitate 5G coverage in remote areas. Regarding the territories with coverage, a large part of the population does not make effective use of the technological tools associated with IoT, causing a high level of nonformalization and low use of these tools [95]. In that sense, the availability of the 5G/IoT service will not be sufficient if people are not properly trained to take advantage of it.

Table 6 provides an overview of the applications of 5G as a communication scenario in the use cases of health, agriculture, education, and public safety. These sectors are considered in this document as their relevance has been highlighted in Colombia’s National Development Plan. In addition, some of them are part of some pilot tests and initiatives for the future deployment of 5G in this country.

## 4. Conclusions

Mobile networks have changed the way people interact, especially when it comes to connecting to the Internet. The same is true for governments, businesses, and organizations in the way they make decisions and deliver services. Colombia is no stranger to this panorama, and the indicators related to this sector are proof of this. For this reason, the national government has ordered the switch-off of 2G networks and their transition to 4G by the end of 2022, mainly benefiting rural areas. It should be noted that there was no evidence of interest on the part of operators to make this full transition in the most isolated areas without imposition by the regulator, possibly due to a lack of economic incentives or benefits. Moreover, if the commitments made in this regard as a result of the 2019 auction are met, there will still be a significant percentage of the population without access to the service.

As for 5G, it is arguably one of the biggest milestones of the last decade in wireless communications, moving from being an expectation to become a reality, with a replacement even being sought with the projected 6G in 2030. Although the first 5G systems were deployed in 2018, the first commercial 5G infrastructure is not yet in Colombia and there is no definitive official date for its deployment and implementation. However, there is a roadmap developed by the MinICT in collaboration with other state agencies and other sector actors, which will allow for the creation of the first private operators’ networks in the short term. The first step in this direction is the allocation of radio spectrum use permits for pilot tests, the main objective of which is to gather information for proper planning. Other actions are equally or more relevant, such as the 4G spectrum auction held in 2019 and the expansion of 4G coverage in a large part of the Colombian territory. Its importance lies in the fact that these networks will be the basis for the first phase of 5G deployment in NSA mode, although it is important to note that it will not offer all the benefits included in the SA architecture, but will allow for easy and rapid deployment at a lower cost. Therefore, before thinking about the mass roll-out of 5G in SA, the central government and TNSPs should focus on the transition from 2G and 3G to 4G and bring this type of network to populations that currently have no mobile Internet service. In turn, these policies will reduce the huge digital divide that exists between more populated and developed urban areas and rural or geographically isolated areas.

Although spectrum management in Colombia is harmonized with international standards, in the authors’ opinion there are obvious shortcomings in the massification of 4G and the implementation of 5G in Colombia. First and foremost is the lack of defined and available spectrum for 5G-only use, which prevents TNSPs from offering the service to users. Another relevant factor is the slow time between auctions, which does not correspond to technological needs and the evolution of mobile communications. Finally, the current spectrum caps for mobile Internet services prevent new operators from entering the market, unless they do so as MVNOs. For this reason, Colombia needs to take steps to reduce spectrum allocation times and increase the current caps.

However, spectrum allocation is not enough: additional policies and strategies need to be adopted, such as incentivizing and allowing the use of bands above 20 GHz, for example, in pilot tests. Even though several TNSPs were interested in this type of frequency, the MinICT only granted permits in the 587 MHz and 3500 MHz bands, delaying the possibility of obtaining relevant information on signal behavior in the centimeter and millimeter bands in urban and suburban scenarios in Colombia. Pilot tests on high frequencies not currently used in mobile services will provide more information on key elements such as propagation and interference with services operating in adjacent bands. Importantly, auctions in other countries have adopted the use of these spectrum bands, demonstrating their importance in the future deployment of 5G, especially in urban or densely populated scenarios. It is also mandatory to know as soon as possible the results of the tests carried out on permits issued in 2020. At the end of July 2022, two years have passed since the authorizations were granted and there are still no official publications on the subject. This information will allow TNSPs that do not benefit from spectrum use permits for pilot testing to use the data to properly structure their 5G investment proposals, including in the future spectrum auction.

On the user side, individuals and businesses or sectors that want to adopt 5G will be forced to replace their existing equipment or purchase new technology if they want to realize the full potential of this technology. To this end, the Colombian state will have to generate standards and adopt policies that encourage the updating or acquisition, not only of terminal equipment but also of other tools such as IoT, DB, and ML.

All the above discussion leads us to conclude that there is currently no favorable scenario for 5G deployment in Colombia before the end of 2022. Regulators and operators should therefore focus their efforts on strengthening and expanding 4G coverage in the short term while the necessary steps towards the 5G scenario are put in place.

## Figures and Tables

**Table 1:** Advantages and disadvantages of different generations of mobile telephony. Source: [6,7,8,9,10].

Generation	Advantages	Disadvantages
1G	- Low energy consumption. - Ease of deployment.	- Low speed and limited services. - Analogue modulation. - Only voice services are allowed. - Lack of standard. - High costs of mobile devices. - Low coverage.
2G	- Digital modulation - Encryption of communications. - Higher spectral efficiency compared to 1G. - Offers additional services such as SMS (short message service) or MMS (multimedia messaging service).	- A large number of standards. - Communication failures in the early stages of deployment caused by digitization.
3G	- Higher-quality voice and data transmission. - Allows access to the Internet. - Offers multimedia services. - Voice over IP.	- Costly infrastructure. - Cell breathing effect.
4G	- Higher spectral efficiency. - Higher bandwidth. - High transmission speeds. - Increased privacy and security. - All IP.	- High power consumption. - Hardware complexity.
5G	- Offers the highest speed rates among all generations. - High bandwidth. - High security. - Improved Quality of Service (QoS). - Low latency. - Facilitates IoT implementation.	- High terminal equipment costs. - High infrastructure costs. - The use of millimeter bands poses coverage problems.

**Table 2:** Information selection criteria applied in search strings.

Filters	Selection Criteria Applied
Document type	Conference paper, article, review, book chapter, conference review, book
Year	2017–2022
Publication stage	Final
Source type	Journal, conference proceedings, book series, book
Language	English

**Table 3:** Summary of spectrum allocations by auction for mobile telephony services in Colombia. Source: [54].

Year	Frequency (MHz)	Amount of Spectrum Assigned	Allocation Value (USD)	Supplier
2010	2500–2690	50 MHz	41.164.757	UNE
2011	1850–1852.5 1930–1932.5	5 MHz	16.099.999	Colombia Móvil S.A. E.S.P. (Tigo)
	1852.5–1855 1932.5–1935	5 MHz	16.099.999	Comunicación Celular S.A. (Claro)
	1867.5–1870 1947.5–1950	5 MHz	15.900.000	Telefónica móviles Colombia S.A. (Movistar)
	1885–1887.5 1965–1967.5	5 MHz	15.900.000	
	1887.5–1890 1967.5–1970	5 MHz	15.900.000	
2013	1710–1725 2110–2125	30 MHz	55.987.819	Avantel S.A.S.
	1725–1740 2125–2140	30 MHz	103.103.657	Telefónica móviles Colombia S.A. (Movistar)
	1740–1755 2140–2155	30 MHz	101.983.901	Unión Temporal Colombia Móvil ETB
	2525–2540 2645–2660	30 MHz	62.516.732	Comunicación Celular S.A. (Claro)
	2555–2570 2675–2690	30 MHz	37.436.499	Direct TV Colombia Ltd.a
	2575–2615	40 MHz	40.410.795	
2014	835.02–844.98 846.51–848.97 880.02–889.98 891.51–893.97	25 MHz	145.265.970 (One-off payment for the 850 MHz and 1950 MHz bands)	Colombia Telecomunicaciones S.A. E.S.P. (Movistar Colombia)
	1875.0–1882.5	15 MHz		
	1955.0–1962.5			
	824.04–825 825.03–834.99 845.01–846.08 869.04–870 870.03–879.99 890.01–891.48	25 MHz	145.265.970 (One-off payment for the 850 MHz and 1950 MHz bands)	Comunicación Celular S.A. (Claro)
	1850–1852.5			
	1855–1860	15 MHz		
	1935–1940			
2019	703–713 758–768	20 MHz	285.673.904	Colombia Móvil S.A. E.S.P. (Tigo)
	713–723 768–778	20 MHz	451.064.060	
	723–733 778–788	20 MHz	285.673.904	Partners Telecom Colombia S.A.S. WOM
	733–743 788–798	20 MHz	285.450.582	Comunicación Celular S.A. (Claro)
	2515–2520 2635–2640	10 MHz	88.154.918	Partners Telecom Colombia S.A.S. WOM
	2520–2525 2640–2645	10 MHz	52.165.481	
	2540–2545 2660–2665	10 MHz	50.822.399	Comunicación Celular S.A. (Claro)
	2545–2550 2665–2670	10 MHz	59.487.802	
	2550–2555 2670–2675	10 MHz	81.535.840	

**Table 4:** Companies offering mobile telecommunications services in Colombia. Source: [59].

Company Providing the Service	Spectrum Use Permit	Virtual Operator
Avantel S.A.S.	✓	✗
Colombia Móvil S.A. E.S.P (Tigo)	✓	✗
Colombia Telecomunicaciones S.A. E.S.P. (Movistar)	✓	✗
Comunicación Celular S.A. COMCEL S.A.	✓	✗
Partners Telecom Colombia S.A.S	✓	✗
Mercanet S.A.S	✗	✓
Empresa Colombiana de Procesos Tecnológicos, Tecnología y Comunicaciones S.A.	✗	✓
Empresa de Telecomunicaciones de Bogotá S.A. E.S.P.	✗	✓
Virgin Mobile Colombia S.A.S.	✗	✓
Nesh Móvil S.A.S.	✗	✓
Setroc Mobile Group S.A.S.	✗	✓
Almacenes Éxito Inversiones S.A.S.	✗	✓
Logística Flash Colombia S.A.S.	✗	✓
Suma Móvil S.A.S	✗	✓

**Table 5:** Companies with approved permits to use radio spectrum for 5G pilots. Source: [35].

Applicant	Frequency Band
Colombia Telecomunicaciones S.A. E.S.P.	3500 MHz–3600 MHz
Comunicación Celular S.A. Comcel S.A.	3500 MHz–3600 MHz
Empresa de Telecomunicaciones de Bogotá S.A. E.S.P.	3500 MHz–3600 MHz
ITICS S.A.S.	3500 MHz–3600 MHz
Xiro Investment Group SAS	3300 MHz–3400 MHz and 587 MHz–592 MHz

**Table 6:** Overview of examples of applications using 5G.

Sector	Summary of Implementation	References
e-health	Transmission of high-definition video and medical images between medical centers and with patients.	[87]
	Classification and remote monitoring of patient’s condition using neural networks.	[96]
	Monitoring and recommending actions to patients in a mobile context.	[97]
Agriculture	Remote control of the irrigation system.	[98]
	A platform for calculation, analysis, and sowing guidance by integrating agronomy, BD, IoT, and 5G.	[99]
	Inspection and early warning robot for greenhouses.	[100]
Education	High-speed mobile network based on open source for virtual education.	[101]
	Model for the development of music education in universities.	[102]
	Video surveillance system based on a wireless sensor network for education and multimedia teaching in classrooms.	[103]
Safety and security	Smart mobile application for police security at large events.	[104]
	UAV-assisted tracking and reconnaissance system.	[105]
	Real-time monitoring of conditions, safety, and privacy on motorways.	[106]

## Data Availability

No new data were created.
